# Allocentric Spatial Memory Testing Predicts Conversion from Mild Cognitive Impairment to Dementia: An Initial Proof-of-Concept Study

**DOI:** 10.3389/fneur.2016.00215

**Published:** 2016-12-01

**Authors:** Ruth A. Wood, Kuven K. Moodley, Colin Lever, Ludovico Minati, Dennis Chan

**Affiliations:** ^1^Department of Medicine, Brighton and Sussex Medical School, Falmer, UK; ^2^Sainsbury Wellcome Centre for Neural Circuits and Behaviour, University College London, London, UK; ^3^Department of Psychology, University of Durham, Durham, UK; ^4^U.O. Direzione Scientifica, Fondazione IRCCS, Istituto Neurologico Carlo Besta, Milan, Italy; ^5^Centro Interdipartimentale Mente/Cervello (CIMeC), Università di Trento, Trento, Italy; ^6^Department of Clinical Neurosciences, University of Cambridge, Cambridge, UK

**Keywords:** Alzheimer’s disease, mild cognitive impairment, spatial memory, hippocampus, Four Mountains Test, dementia

## Abstract

The hippocampus is one of the first regions to exhibit neurodegeneration in Alzheimer’s disease (AD), and knowledge of its role in allocentric spatial memory may therefore aid early diagnosis of AD. The 4 Mountains Test (4MT) is a short and easily administered test of spatial memory based on the cognitive map theory of hippocampal function as derived from rodent single cell and behavioral studies. The 4MT has been shown in previous cross-sectional studies to be sensitive and specific for mild cognitive impairment (MCI) due to AD. This report describes the initial results of a longitudinal study testing the hypothesis that allocentric spatial memory is predictive of conversion from MCI to dementia. Fifteen patients with MCI underwent baseline testing on the 4MT in addition to CSF amyloid/tau biomarker studies, volumetric MRI and neuropsychological assessment including the Rey Auditory Verbal Learning Test (RAVLT) and Trail Making Test “B” (TMT-B). At 24 months, 9/15 patients had converted to AD dementia. The 4MT predicted conversion to AD with 93% accuracy (Cohen’s *d* = 2.52). The predictive accuracies of the comparator measures were as follows: CSF tau/β-amyloid_1–42_ ratio 92% (*d* = 1.81), RAVLT 64% (*d* = 0.41), TMT-B 78% (*d* = 1.56), and hippocampal volume 77% (*d* = 0.65). CSF tau levels were strongly negatively correlated with 4MT scores (*r* = −0.71). This proof-of-concept study provides initial support for the hypothesis that allocentric spatial memory testing is a predictive cognitive marker of hippocampal neurodegeneration in pre-dementia AD. The 4MT is a brief, non-invasive, straightforward spatial memory test and is therefore ideally suited for use in routine clinical diagnostic practice. This is of particular importance given the current unmet need for simple accurate diagnostic tests for early AD and the ongoing development of potential disease-modifying therapeutic agents, which may be more efficacious when given earlier in the disease course. By applying a test based on studies of hippocampal function in rodents to patient populations, this work represents the first step in the development of translatable biomarkers of hippocampal involvement in early AD for use in both animal models and human subjects.

## Introduction

The anticipated arrival of disease-modifying drugs for the treatment of Alzheimer’s disease (AD) places increased emphasis on the need to identify AD in its earliest stages, when such treatments may have greatest benefit in delaying or preventing the onset of dementia. It is now accepted that AD presents as mild cognitive impairment (MCI) in its prodromal stages (1), but MCI can also be caused by non-AD disorders with lower dementia risk, including non-neurodegenerative conditions such as anxiety. Identification of MCI on clinical grounds alone cannot discriminate between the various differing underlying etiologies (2, 3). The high prevalence of memory impairment in the aging population [in a US study, 27% of people over 65 years reported memory decline (4)] amplifies the difficulty of accurately differentiating MCI due to AD and highlights the need for diagnostic tests that are not only sensitive and specific for pre-dementia AD but are also non-invasive and usable in routine clinical diagnostic practice.

This need is not met by current tests. Screening tests of global cognitive function of the kind typically used in primary care, such as the Mini-Mental State Examination (MMSE), have low diagnostic specificity and are poor predictors of conversion to dementia (5, 6). Tests of specific cognitive domains, such as episodic memory or attention, have higher diagnostic accuracy but require training in administration and scoring and are primarily used in specialist clinics (7–9). Biomarker-based tests, such as MRI measurements of hippocampal atrophy, amyloid-PET scanning, or CSF studies of amyloid/tau, have higher predictive accuracy (10–12), but the high cost, limited availability, and invasive nature of some tests preclude their usage in routine clinical practice.

This need may be met by a hippocampus-dependent test of allocentric spatial memory. Neuropathological studies have shown the hippocampus to be one of the first brain regions affected in AD (13), and there is extensive evidence that the hippocampus is critically involved in spatial memory, with the demonstration in rodents of spatially related firing activity of hippocampal neurons “place cells” (14) and of a correlation between place cell activity and spatial memory (15). Importantly, in an open arena, hippocampal spatial neurons signal location in an allocentric reference frame. Place cells and boundary cells fire in a viewpoint-independent manner such that a given neuron fires at broadly similar rates whether the subject is, for instance, facing north or south as it moves through the neuron’s locational firing field (16, 17). This signaling of viewpoint-independent location is a defining characteristic of hippocampal spatial representation.

A role for the human hippocampus in spatial memory is supported by pre-surgical depth electrode recordings of place-related firing of hippocampal neurons (18), and functional imaging studies showing activation of the hippocampus during spatial memory tasks (19, 20). Patients with focal hippocampal lesions show disproportionate impairment of spatial memory (21), and several studies have shown that spatial memory is impaired in patients with MCI and AD (22–25). However, to date, the ability of spatial memory testing to predict conversion from MCI to AD dementia has not been evaluated.

A variety of differing behavioral paradigms have been used to test spatial memory in humans. These include the Hidden Goal Task (26), which was designed as a human analog of the Morris water maze used for rodent studies (27) and assesses memory for hidden locations within a three meter diameter, circular, velvet arena, as well as a variety of tests of spatial navigation and memory using desktop-based virtual reality (VR) paradigms (22, 28). However, these paradigms have operational limitations that render them unsuitable for use as diagnostic tests for prodromal AD in routine clinical practice. The space requirements of the Hidden Goal Task effectively precludes its usage beyond research labs with dedicated testing arenas, whereas the various VR tests used to date require significant tester input and have been associated with nausea and disorientation when applied to older individuals with early AD (29).

The 4 Mountains Test (4MT) is an easily administered test of allocentric spatial memory, which builds upon the defining characteristic of hippocampal spatial representation mentioned above in discussing rodent spatial neurons: the signaling of viewpoint-independent location. Essentially, the task requires that the participant correctly identifies a previously seen location, though the viewpoint the location is seen from has shifted from sample to test. The 4MT is specifically designed to be resistant to non-spatial strategies (30). The 4MT can be applied in paper form, with or as an iPad app, and requires little in the way of tester training or instructions (a video example of test administration is viewable *via* the open access article by Chan et al. (31)). The brevity of the test (around 10 minutes for the paper version and around 8 minutes for the app) is comparable to the MMSE (Mini Mental State Examination Test) (5), which is widely used worldwide as a short screening test for cognitive impairment but a poor predictor of progression from MCI to dementia (6). Following the initial use of this test to demonstrate that patients with focal hippocampal damage were selectively impaired on spatial memory testing, with relative preservation of spatial perception and non-spatial memory (30), performance on the 4MT has been found to discriminate between AD and non-AD dementia (29, 32) and between MCI patients with and without CSF biomarker evidence of underlying AD (33).

Following on from these cross-sectional studies, this initial longitudinal study used the 4MT to test the primary hypothesis that allocentric spatial memory test performance is predictive of conversion from MCI to AD dementia. Additionally, the secondary hypothesis that spatial memory is a behavioral marker of hippocampal neurodegeneration in early AD was tested by correlating 4MT scores with levels of CSF total tau, representing a biomarker of neurodegeneration in AD.

## Materials and Methods

### Subjects

Fifteen patients with multiple domain MCI, all of whom presented with memory impairment, were recruited from the Cognitive Disorders Clinic, Hurstwood Park Neurological Centre, Haywards Heath, West Sussex, and from the East Sussex Memory Assessment Service. The baseline cross-sectional data on 13/15 MCI patients have been reported by Moodley et al. (33), and all baseline data are summarized in Table 1. MCI was diagnosed by a neurologist (Kuven K. Moodley and Dennis Chan) in accordance with the Petersen criteria (34). Initial screening blood tests were undertaken to exclude reversible causes of cognitive impairment, such as vitamin B12 deficiency and thyroid dysfunction. Subjects were excluded from the study if they had depression, other psychiatric diagnoses, a significant vascular lesion load on MRI and/or a Hachinski Ischemic Score >4 (35). The degree and extent of objective cognitive impairment was established at baseline in clinic using the Addenbrooke’s Cognitive Examination – Revised (36), which includes the MMSE (5), or the Queen Square Screening Test for Cognitive Deficits (©EK Warrington, 2003) plus the MMSE.

**Table 1:** **Demographic variables and candidate predictors of conversion**.

Demographic or predictor variable	MCI – converters (mean ± SE)	MCI – non-converters (mean ± SE)	Difference statistic	*p*-value*	Cohen’s *d* (effect size)
Gender	2 F:7 M	2 F:4 M	X^2^_1_ = 0.227	0.63	–
Age	71.7 ± 3.0	65.2 ± 3.4	T_13_ = 1.41	0.18	0.75
Years of education	12.1 ± 0.7	11.0 ± 0.5	T_13_ = 1.19	0.26	0.67
NART estimated IQ	106.6 ± 3.9	114.7 ± 3.4	T_10_ = 1.50	0.16	0.90
4MT score	5.56 ± 0.71	10.17 ± 0.60	T_13_ = 4.60	**0.0005**	2.52
TMT-B (s)	132.55 ± 14.98	80.78 ± 8.36	T_12_ = 2.74	**0.018**	1.56
RAVLT score	2.38 ± 0.62	3.33 ± 0.14	T_12_ = 0.79	0.45	0.41
MMSE score	27.89 ± 0.42	27.33 ± 0.21	T_13_ = 1.01	0.33	0.58
Total hippocampal volume (% of total intracranial volume)	0.509 ± 0.037	0.577 ± 0.45	T_11_ = 1.18	0.26	0.65
CSF total tau:β-amyloid_1–42_	3.57 ± 0.92	0.45 ± 0.09	T_11_ = 3.12	**0.001**	1.81

*MCI, mild cognitive impairment; 4MT, Four Mountains Test; TMT-B, Trail Making Test B; RAVLT, Rey Auditory Verbal Learning Test; MMSE, Mini Mental State Examination*.

*Uncorrected p-values.

*Bold font signifies p-values that are statistically significant at the p < 0.05 level*.

As part of their diagnostic work-up 13/15 MCI subjects had CSF tested for AD biomarkers (CSF β-amyloid_1–42_, CSF tau, CSF tau/β-amyloid_1–42_ ratio) using industry standard ELISA assay kits (Innotest, Innogenetics, Ghent, Belgium) in accordance with the CSF collection protocol of the CSF sub-study of the Alzheimer’s disease Neuroimaging Initiative (ADNI) (37). The 13/15 subjects had MRI scans with T1-weighted volumetric MRI data acquired on a 1.5T Siemens Avanto scanner based at the Clinical Imaging Sciences Centre, Brighton and Sussex Medical School using a magnetization-prepared rapid-acquisition gradient-echo sequence to generate voxels of 1 mm × 1 mm × 1 mm. Total hippocampal volumes, corrected for total intracranial volume, were measured using the FSL(version 5.0)/FIRST tool (FMRIB, Oxford Centre for Functional Magnetic Resonance Imaging of the Brain, Oxford, UK) (38). In those instances where CSF and MRI investigations were not undertaken, this was due to patient preference.

The study was undertaken in accordance with the Declaration of Helsinki and all participants provided written informed consent. Ethical approval was obtained from the UK Research Ethics Committee South East Coast – Brighton and Sussex (references 10/H1107/23 and 13/LO/0277).

### The 4 Mountains Test

**Figure 1:**
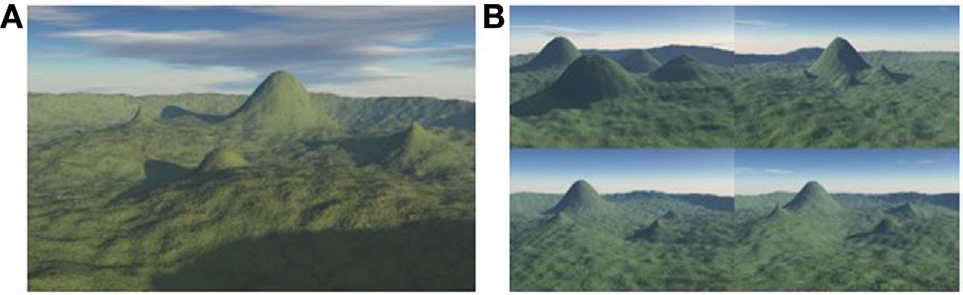
**The 4 Mountains Test**. **(A)** A landscape containing four mountains is presented for 8 seconds and then removed. **(B)** After a 2 second delay, this landscape is re-presented, but from a rotated viewpoint, with three additional “foil” landscapes, in a delayed match-to-sample paradigm (the correct response is the bottom right image).

A full description of the 4MT is provided by Hartley et al. (30). The spatial memory test involves presentation of computer-generated landscapes, each containing images of four mountains arranged around the center of the landscape, in the form of printed color images within an A4-sized booklet. For each test item, a sample image is presented for eight seconds. After a two second delay, this image is re-presented, but from a different viewpoint, alongside three foil images of landscapes with differing topography (Figure 1). Fifteen test items were shown in all, and the total test duration was approximately ten minutes. Participants completed the 4MT alongside additional neuropsychological tests at entry into the study, with all testing undertaken during a single sitting.

### Baseline Neuropsychological Testing

Subjects completed a battery of baseline neuropsychological tests, which included the National Adult Reading Test (NART) (39) to obtain an estimation of premorbid IQ in line with numerous other studies of cognitive function in clinical cohorts, and subtests of the Visual Object and Space Perception battery (40) for assessment of visual perception. The neuropsychological test battery also included two tests considered to be sensitive to early AD (1) and thus effective predictors of progression from MCI to AD dementia (8, 41). These are the Rey Auditory Verbal Learning Test [RAVLT; (42)], a test of episodic memory, and the Trail Making Test “B” (TMT-B) (43), a test probing several cognitive domains including attention, speed of information processing, and executive function. One subject declined to complete the tests outlined above due to anxiety.

### Determination of Conversion Status

Conversion status was determined 24 months after study enrollment by a neurologist (Kuven K. Moodley and Dennis Chan) in clinic, blinded to the baseline 4MT score, with the diagnosis of AD dementia made in accordance with the 2011 McKhann criteria (44). In addition to the presence of cognitive or behavioral symptoms involving at least two cognitive domains, conversion to AD dementia from MCI was based on decline in cognitive function on objective testing, new impairment in activities of daily living and loss of functional independence, representing changes from the time of the previously attributed MCI diagnosis.

### Comparison of 4MT Performance with Other Measures

The ability of the 4MT to predict conversion from MCI to dementia was compared with five other measures. In addition to the RAVLT (delayed free recall score) and TMT-B (completion time), comparisons were made with the MMSE score, total hippocampal volume, and CSF tau/β-amyloid_1–42_ ratio.

The rationale for these additional comparisons is as follows. While systematic reviews have shown that the predictive ability of the MMSE is low (6), it is part of the Preclinical Alzheimer Cognitive Composite (45) that is approved by the FDA for use as an outcome measure in trials of treatments aiming to delay progression from MCI to dementia. Furthermore, the MMSE is used widely as a cognitive screening test in UK memory services, notably in primary care, and as such the MMSE score has a bearing on the clinical management of patients presenting with MCI. Total hippocampal volume and CSF tau/β-amyloid_1–42_ ratio are included as comparators in view of their status as AD biomarkers included within research criteria for the diagnosis of MCI due to AD (1).

These five comparator measures were analyzed as both continuous (Table 1) and binary (Table 2) variables.

**Table 2:** **Binary classification using candidate predictor variables**.

	Criterion for positive test (see methods)	Prevalence (of converter positive cases in sample, %)	Sensitivity (%)	Specificity (%)	PPV:NPV (%)	Accuracy (%)	Youden’s *J*	AUC ± SE	AUC *p*-value (0.5 AUC = *H*_0_)
4MT score	≤8	60 (*n* = 15)	100	83.3	90.0:100	93.3	0.833	0.981 ± 0.022	**<0.0001**
TMT-B (s)	>102.6	57.1 (*n* = 14)	62.5	100	100:66.7	78.6	0.625	0.875 ± 0.094	**0.0001**
RAVLT score	≤3	57.1 (*n* = 14)	75.0	50.0	66.7:60.0	64.3	0.250	0.604 ± 0.168	0.54
MMSE score	≤27	60 (*n* = 15)	44.4	33.3	50.0:28.6	40.0	−0.222	0.370 ± 0.140	>0.5*
Total hippocampal volume (% of TIV)	≤0.570	53.8 (*n* = 13)	85.7	66.7	75.0:80.0	76.9	0.524	0.714 ± 0.163	0.19
CSF total tau:β-amyloid_1–42_	>0.821	53.8 (*n* = 13)	85.7	100	100:85.7	92.3	0.857	0.905 ± 0.100	**0.0001**

*PPV, positive predictive value; NPV, negative predictive value; AUC, area under curve; TIV, total intracranial volume*.

*An exact p-value is not given here as the ROC curve for MMSE was below the chance line.

*Bold font signifies p-values that are statistically significant at the p < 0.05 level*.

### Statistical Analysis

Data were analyzed using MedCalc Statistical Software version 15.6.1 (MedCalc Software bvba, Ostend, Belgium, 2015). For all continuous data, normality was assessed using normal probability plots and Shapiro–Wilk testing, and Levene’s test was applied to ensure homogeneity of variances. No corrections were necessary. Cohen’s *d* provided a measure of effect size, calculated by dividing the difference between the group means by their pooled SD (√[(StDevConverters^2^ + StDevNon-Converters^2^)/2]).

The threshold criteria for binary classification (MCI converters vs. MCI non-converters) for each of the comparators were selected by maximizing Youden’s index *J* (Table 2), where Youden’s *J* = sensitivity + specificity − 1. The SE of the area under the curve (AUC) and the probability value of the AUC under the null hypothesis of AUC = 0.5 were calculated according to the method of DeLong et al. (46).

## Results

### Demographics

There were no statistically significant differences between MCI converters and MCI non-converters in terms of age [71.7 ± 3.0 years (mean ± SE) vs. 65.2 ± 3.4, *p* = 0.18], years of education (12.1 ± 0.7 vs. 11.0 ± 0.5, *p* = 0.26), gender (2 F:7 M vs. 2 F:4 M, *p* = 0.634), or NART estimated IQ (106.6 ± 3.9 vs. 114.7 ± 3.4, *p* = 0.16). An ANCOVA of the 4MT score vs. converter status, with age and IQ as covariates, showed that their effect on conversion status was non-significant (*p* = 0.223, *p* = 0.332, respectively). The effect of 4MT score on conversion status remained significant even after accounting for group differences in age and IQ.

### Correlation of 4MT Score with CSF AD Biomarkers

**Figure 2:**
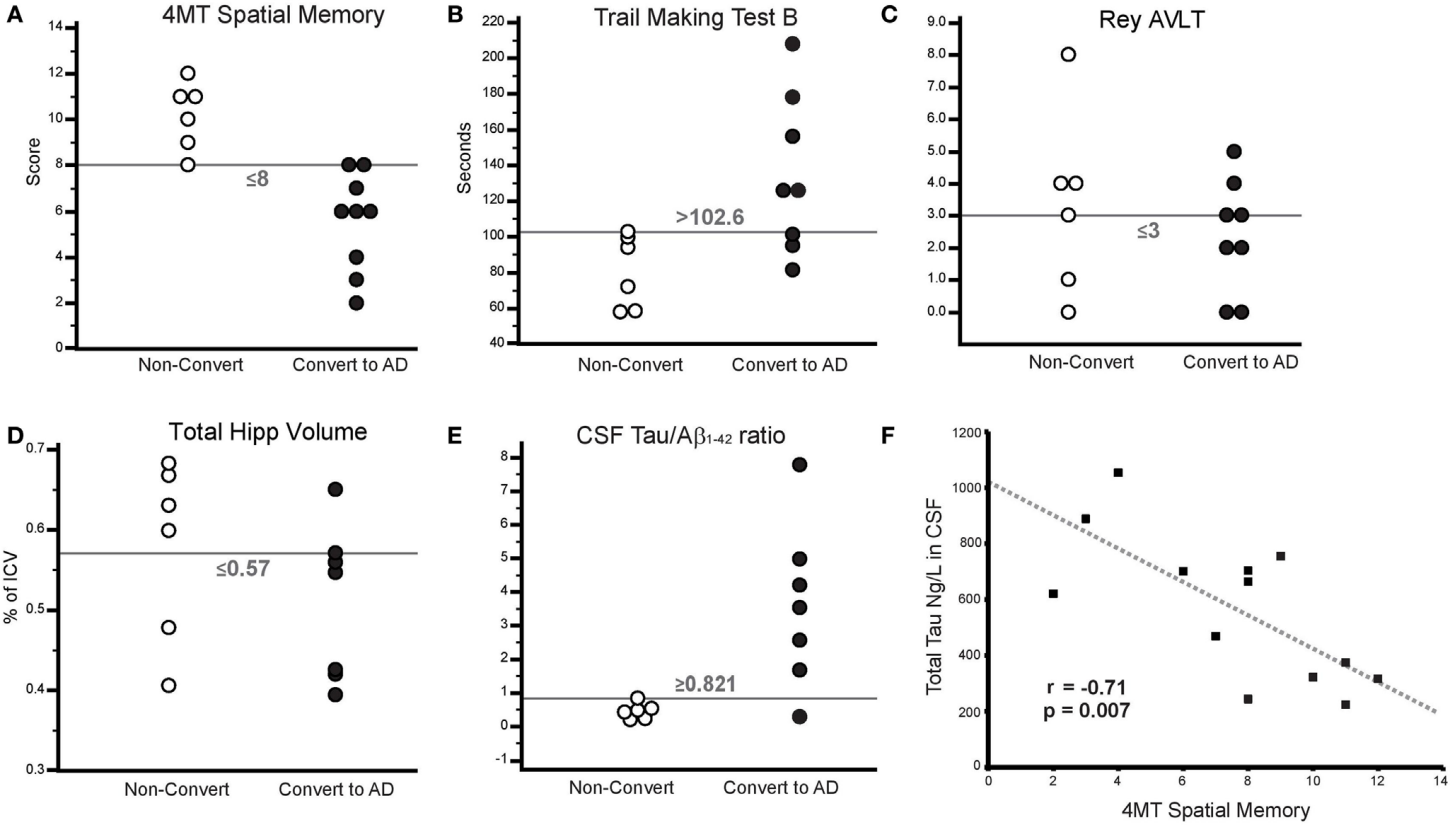
**(A–E)** Threshold plots illustrating the differences in scores/measures between converters and non-converters. **(A)** 4 Mountains Test (4MT) spatial memory score, **(B)** Trail Making Test B (TMT-B) time (s), **(C)** Rey Auditory Verbal Learning Test (RAVLT) score (Delayed Free Recall), **(D)** total hippocampal volume (as % of total intracranial volume), and **(E)** CSF tau:β-amyloid_1–42_. **(F)** Scatterplot illustrating the relationship between CSF total tau and 4MT score. Gray dashed line indicates line of best fit.

4MT scores were strongly negatively correlated with the levels of CSF total tau (*r* = −0.71, *p* = 0.007; Figure 2F) but did not correlate with the levels of CSF Aβ_1–42_ (*r* = 0.5, *p* = 0.08). CSF tau levels were negatively correlated with total hippocampal volume (*r* = −0.59, *p* = 0.04) in keeping with previous studies (47, 48), but after controlling for hippocampal volume, CSF tau still robustly predicted 4MT spatial memory score (*n* = 12, partial *r* = −0.84, *p* = 0.001).

Thirteen subjects were available for the 4MT vs. hippocampal volume correlation. An *r* value ≥0.56 would be required for significance in a two-tailed test where *n* = 13. The correlation coefficient obtained in this sample, *r* = 0.43, is therefore not statistically significant. However, it is worth noting that this value is similar to that observed in a much larger sample of MCI and AD patients (33).

### Accuracy of 4MT and Comparator Variables in Predicting Conversion to Dementia

At 24-month follow-up, 9/15 MCI patients converted to AD dementia, with the remaining 6/15 patients having unchanged diagnoses of MCI. MCI converter and non-converter groups differed significantly on 4MT (lower scores in converters, *p* = 0.0005, Cohen’s *d* = 2.52), TMT-B (longer completion time in converters, *p* = 0.018, Cohen’s *d* = 1.56), and in terms of CSF tau/β-amyloid_1–42_ ratios (lower ratios in converters, *p* = 0.001, Cohen’s *d* = 1.81) (Table 1; Figures 2A,B,E). The two groups did not differ significantly in terms of MMSE score, RAVLT delayed free recall score or total hippocampal volume (all *p*-values ≥ 0.33, Table 1; Figures 2C,D). ROC curve analysis for the study predictor variables showed that the AUC was significantly above chance for 4MT score (AUC = 0.981, *p* < 0.0001), CSF tau/β-amyloid_1–42_ ratio (AUC = 0.905, *p* = 0.0001), and TMT-B time (AUC = 0.875, *p* = 0.0001) (Table 2). MMSE, RAVLT, and total hippocampal volume did not predict conversion (all *p*-values ≥ 0.19, Table 2; Figures 2C,D).

Only two measures were associated with overall classification accuracies and Youden’s *J* values above 80%, namely the 4MT score (93%, 0.833) and CSF tau/β-amyloid_1–42_ ratios (93%, 0.857) (Table 2; Figures 2A,E).

## Discussion

Spatial memory is impaired in patients with mild dementia due to AD (24, 25, 32, 49), and more recent work has demonstrated that impairment is already evident in the pre-dementia stages of AD (33, 50). Both allocentric and egocentric spatial memory have been observed to be defective in patients with amnestic MCI (22–25), with some evidence that allocentric spatial memory, dependent on hippocampal function, is more impaired in these patients than egocentric spatial memory, which is more dependent on striatal function (51).

No studies to date have demonstrated that performance on a spatial memory task can predict conversion from MCI to AD dementia. Given the prevalence of memory impairment in the aging population and the difficulty of distinguishing MCI due to AD from other causes of memory impairment (such as depression or anxiety) on clinical grounds alone, the identification of a test with high predictive value would have major benefits for clinical practice on a large scale.

The 4 Mountains Test (4MT) of allocentric spatial memory has been shown in a previous cross-sectional study to differentiate MCI due to AD (32, 33). This initial longitudinal study of 15 MCI patients followed up over 24 months provides early support for the hypothesis that allocentric spatial memory testing is an accurate predictor of conversion from MCI to AD dementia. Performance on the 4MT predicted conversion with a classification accuracy of 93%, superior to the predictive accuracy of cognitive tests widely used in clinical and research practice for the diagnosis of MCI, with a 64% accuracy observed with the RAVLT and a 79% accuracy associated with the TMT-B. Given its widespread usage in clinical practice worldwide, a comparison was also made with the MMSE, and the low predictive accuracy (40%) associated with this test in the current study is consistent with the findings of a recent systematic review (6), which concluded that the MMSE was a poor predictor of conversion.

Further comparison with the predictive accuracy of AD biomarkers was undertaken. Total hippocampal volume, widely used as an outcome measure in treatment trials aimed at delaying the progression from MCI to AD dementia, was associated with an accuracy of 77%. The 92% predictive accuracy of the CSF tau/β-amyloid_1–42_ ratio is similar to the 93% accuracy of the 4MT, while the positive and negative predictive values of the CSF tau/β-amyloid_1–42_ ratio of 100 and 86%, respectively, bear comparison with figures of 81 and 96% from a previous longitudinal study (52).

CSF total tau, considered to be a measure of tau release from dying or dysfunctional neurons containing neurofibrillary tangles, is used as a biomarker of neurodegeneration in AD and levels of CSF total tau are negatively correlated with performance on the RAVLT and semantic fluency tests (47, 53). While correlations are also observed with hippocampal volume (47), this is the first study to report a correlation between CSF total tau and performance on a hippocampus-dependent spatial memory task. The strength of the correlation (*r* = −0.71) suggests that testing of allocentric spatial memory may represent a behavioral marker of tau-related hippocampal neurodegeneration. This has increased significance in light of the current practice of using β-amyloid biomarkers for diagnosing AD in its pre-dementia stages or as screening tests for clinical trials, in the form of CSF β-amyloid_1–42_ or amyloid-PET scanning. The recent observation that a third of cognitively healthy older individuals with positive CSF amyloid biomarkers did not exhibit any cognitive decline over 9-year follow-up (54) illustrates the limitations associated with β-amyloid biomarkers and the importance of markers of neurodegeneration in predicting cognitive decline in individuals at risk of developing dementia.

In addition to the above, these initial findings have further implications for experimental medicine and clinical practice. First of all, the successful application to clinical diagnostic practice of a test based on a theory of hippocampal function derived from single cell and behavioral studies in rodents (55) helps bridge the divide between basic and clinical neuroscience and represents a significant step forward in the development of translatable behavioral markers of early AD. Second, the brief duration of the 4MT, combined with its ease of application and scoring, ensures that this test is ideally suited for use as a diagnostic test for pre-dementia AD not only in specialist clinics but also in non-specialist diagnostic practice, such as primary care clinics and community-based memory assessment services. These typically represent the first points of medical contact for people with memory decline and the potential value of the 4MT in this context is underscored by the observation in this study (and others) that the MMSE, which is the cognitive test most widely used in non-specialist clinics, is a poor predictor of conversion from MCI to dementia.

The usability and potential for widespread clinical application of the 4MT differentiates this test from other behavioral tests of spatial memory. Alternative tests of allocentric spatial memory, such as the virtual radial maze task (28, 49), the Hidden Goal Task (23) and the Virtual Route Learning Test (29), are based on the same underlying theoretical principles as the 4MT and accordingly have identified spatial behavioral deficits in patients with MCI. However, the complexity of test administration (especially with older adults) and problems with tolerability (e.g., nausea experienced during testing) limits the utility of these tasks in routine diagnostic practice. To date, the accuracy of these tasks for predicting conversion from MCI to AD has not been evaluated, and further studies comparing their prognostic value with that of the 4MT would be of considerable interest.

Several further issues are raised by this initial work. One is that of the incremental added value of the 4MT, above and beyond that of “traditional” cognitive tests. The comparisons with the RAVLT and TMT-B outlined above and in our previous cross-sectional study (33) show a superior diagnostic sensitivity of the 4MT for prodromal AD and a greater predictive ability, and therefore provide first evidence of added prognostic value. These data justify further investigation of this critical issue by application of logistic regression statistical methodologies within currently running large sample size studies.

In this study a 30% annual conversion rate from MCI to dementia was observed which exceeds the 20% annual conversion rate previously reported for MCI with positive CSF AD biomarkers (52). This may reflect the recruitment from a tertiary referral memory clinic, typically associated with higher conversion rates than community-based populations, but may also be a consequence of the small sample size.

It is crucial to reiterate the point that these are preliminary observations. While these findings are sufficient to provide initial proof of concept, the small sample size does not permit any *definitive* conclusions to be drawn. Furthermore, only relatively strong correlations can be detected with this sample size. For instance, while a strong correlation between 4MT score and CSF total tau was identified, the presence of a correlation between 4MT score and hippocampal volume will need to be established in a larger sample.

Nonetheless, this initial dataset has been considered sufficiently robust for the 4MT to be included in the test battery used to evaluate cognitive function in prospective, large scale longitudinal studies of the older population at risk of developing dementia, namely the *n* = 750 PREVENT study (56) and the *n* = 6000 EPAD (European Prevention of Alzheimer’s Disease, http://ep-ad.org) initiative. These studies of asymptomatic at-risk individuals will be complemented by further longitudinal studies of MCI patients whose aims are to determine the predictive accuracy of the 4MT in larger cohorts drawn from community- and hospital-based memory clinics as well as the specificity of this test in determining future conversion to AD and non-AD dementia.

## Conclusion

This study provides the first evidence supporting the hypothesis that testing of allocentric spatial memory is predictive of conversion from MCI to AD dementia. The correlation between CSF total tau and 4MT score indicates that the latter may represent a behavioral marker of hippocampal neurodegeneration. The brevity, non-invasiveness and ease of application of the 4MT means that this test is capable of fulfilling the current unmet need for an accurate test of pre-dementia AD that can be scaled up to address the high diagnostic demand associated with the prevalence of memory impairment in the aging population.

## Author Contributions

DC conceived and designed the study. KM and RW acquired the data and undertook initial data analyses. RW performed the MRI analysis with LM. CL analyzed the remaining data and performed all statistical analyses. RW co-wrote the manuscript with DC, with critical contributions from CL.

## Conflict of Interest Statement

The authors declare that the research was conducted in the absence of any commercial or financial relationships that could be construed as a potential conflict of interest.
